# General Correlated Geminal Ansatz for Electronic Structure Calculations: Exploiting Pfaffians in Place of Determinants

**DOI:** 10.1021/acs.jctc.0c00165

**Published:** 2020-08-17

**Authors:** Claudio Genovese, Tomonori Shirakawa, Kousuke Nakano, Sandro Sorella

**Affiliations:** † SISSA, International School for Advanced Studies, Via Bonomea 265, 34136 Trieste, Italy; ‡ Computational Materials Science Research Team, RIKEN Center for Computational Science (R-CCS), Kobe, Hyogo 650-0047, Japan; § School of Information Science, Japan Advanced Institute of Science and Technology (JAIST), Asahidai 1-1, Nomi, Ishikawa 923-1292, Japan

## Abstract

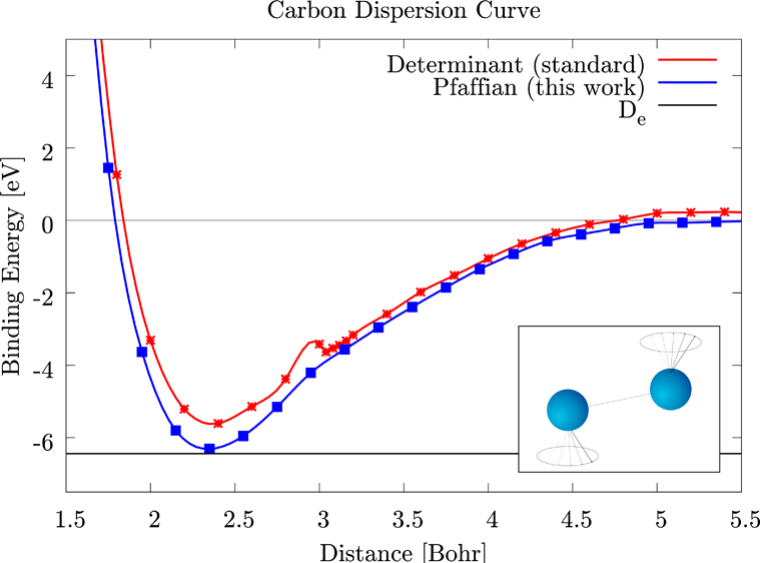

We propose here a single Pfaffian correlated variational ansatz that dramatically improves the accuracy with respect to the single determinant one, while remaining at a similar computational cost. A much larger correlation energy is indeed determined by the most general two electron pairing function, including both singlet and triplet channels, combined with a many-body Jastrow factor, including all possible spin–spin, spin–density, and density–density terms. The main technical ingredient to exploit this accuracy is the use of the Pfaffian for antisymmetrizing a highly correlated pairing function, thus recovering the Fermi statistics for electrons with an affordable computational cost. Moreover, the application of the diffusion Monte Carlo, within the fixed node approximation, allows us to obtain very accurate binding energies for the first preliminary calculations reported in this study: C_2_, N_2_, and O_2_ and the benzene molecule. This is promising and remarkable, considering that they represent extremely difficult molecules even for computationally demanding multideterminant approaches, and opens therefore the way for realistic and accurate electronic simulations with an algorithm scaling at most as the fourth power of the number of electrons.

## 1 Introduction

The accurate determination of the many-electron wave function (WF) has always been a challenging task starting from the early stage of quantum mechanics.^1^ So far, several attempts have been made toward this direction ranging from CCSD(T)^2^ to tensor network^3^ and density matrix renormalization group (DMRG),^4^ up to the very recent breakthrough with the use of machine learning methodologies.^5^ All these schemes pay the price of being computationally demanding, with a computational complexity ranging from a large degree polynomial of the number of electrons to exponential complexity.

Quantum Monte Carlo (QMC) techniques for electronic structure calculations have proven to be very successful in describing the electronic correlation encoded in a many-body WF.^6−8^ In particular, the variational Monte Carlo (VMC)^9−11^ samples the real space electronic configurations of the considered system with a probability distribution given by the WF square, thus providing the efficient evaluation not only of the total energy but also of the expectation values of most commonly used many-body operators. Within VMC, it is possible to improve the description of the ground state (GS) WF by minimization of the total energy expectation value. The WF obtained can be used as it is or further improved by the diffusion Monte Carlo calculation (DMC) method.^10−13^ This technique is a projection algorithm performed statistically using the information on the sign contained in the given WF, dubbed here as a guiding function. In this way, we can considerably improve the description of the GS, projecting on the lowest possible energy WF with the same signs of the guiding WF. In the ideal case of a guiding function that, for every configuration, has the same sign of the GS, the above-described DMC algorithm provides the exact solution.^10,11^

In the framework of QMC, different *ansatzs* are used to approximate the true GS WF, with the purpose to achieve an affordable compromise between the accuracy of the calculation and its computational cost. Though a good representation of the GS can sometimes be achieved with a simple and “cheap” WF, in most cases, the use of a very complicated and computationally demanding *ansatz* is necessary to get a correct answer.

Slater determinants (SDs) are the simplest fermionic WF used for QMC. They provide a single particle picture of the quantum many-body problem, preserving the Pauli principle, that is, the Fermi statistics for electrons. They can be obtained directly from mean-field calculations. Unfortunately in many situations of interest, it is not possible to give a good description of the system in terms of a single SD.^14−17^ In QMC, there are two possible strategies to overcome this problem: the use of a linear combination of different SDs^17−21^ or different *ansatzs* with larger variational freedom.^22,23^ The multideterminant WFs can be systematically improved and in principle can describe exactly every GS with a large enough number of SDs. Unfortunately the number of SDs that has to be taken into account scales exponentially with the number of electrons preventing the calculations on large systems.^24^

The use of pairing function replaces the single particle description of the SD with a richer one in terms of electron pairs. The corresponding WF is a natural extension of the single SD *ansatz* and represents a direct and efficient implementation of the Anderson resonating valence bond (RVB)^25^ theory of many-electron WFs. In particular, it provides a direct description of the singlet and triplet correlations that are absent in the SD. Depending on the definition of the pairing function, qualitatively different WFs can be obtained. They will be described in Section 2, where we will focus also on the technical details required for the calculation. We will introduce the symmetric antisymmetrized geminal power (AGP)^22,26,27^ and the broken symmetry antisymmetrized geminal power (AGPu),^28^ but we will mainly focus on the most general AGP. In the previous literature,^23,29^ it has been indicated as Pfaffian WF, and people have been referring to the AGPs as AGP, but since the AGP (or Pfaffian WF) literally realizes the most general antisymmetrized geminal power, we dub this case with the shortest acronym, that is, AGP. It will be shown that this approach becomes very efficient in combination with an explicit correlation term, known as a Jastrow factor (JF),^14,30,31^ that promotes or penalizes the bonds according to the electronic correlation. As it will be shown later, we have introduced a quite general JF, depending both on spin and electron charges. When it is applied to an AGP without definite spin, it allows its almost complete restoration, mimicking in this way a spin projection operation that, though approximate, is much cheaper than other approaches.^32,33^ Even if the pairing functions cannot be improved systematically, these WFs have a much larger variational freedom than the SDs, nevertheless remaining with a similar computational cost.

If on one hand, for the multideterminant WF, the calculation can be computationally very expensive, on the other hand, for the pairing functions, the optimization of a large number of nonlinear variational parameters can become a serious limitation if not handled efficiently. Indeed, in order to exploit the full potential of these *ansatzes*, it has been fundamental to use the most recent techniques for the calculation of the derivatives and optimization strategies.

In a previous attempt, the AGP WF was used by exploiting only a very small fraction of the large variational freedom of the *ansatz*.^23,29^ The results were not encouraging, and the energies obtained with this *ansatz* did not improve the ones of the AGPs that, in turn, has a lower computational cost. Despite the Pfaffian was no longer used in the electronic system to our knowledge, the experience with lattice models has shown that the AGP WF is able to improve considerably the description of magnetic and correlated systems.^34^ Moreover, the introduction of a powerful JF and the recent results obtained in combination with the AGPs^14,35,36^ encouraged us to look for the unexpressed potential of the full AGP WF.

In this paper, we will compare the results obtained with AGP WF with available state of the art VMC and DMC calculations. In particular, we benchmark our WF on the diatomic molecules with corresponding high spin atoms in the first row of the periodic table, i.e., carbon, nitrogen, and oxygen, and on the benzene. The first ones are systems that, despite their apparent simplicity, represent useful benchmarks for many highly correlated methods.^37−40^ We will show that, with the use of our best WFs, even with a very compact basis set, we are able to achieve an accuracy comparable with the state of the art multideterminant WFs at a computational cost similar to the one of a single SD. Not only the total energies and the dissociation energies are extremely accurate but we also analyzed the magnetic proprieties of these molecules, unveiling the unexpected rich physics behind these systems. Finally we consider the benzene molecule, a system that represents the prototypical example of the RVB theory and thus a fundamental test case for our approach.

## 2 WFs and Procedures

For all calculations we present in this paper, we used the TurboRVB package for QMC calculations.^41,42^ The WFs used for this work are factorized as the product of a fermionic mean field and an explicit bosonic correlation factor. Being Ψ(**X**) the WF of a given configuration **X** = (**r**_1_σ_1_, **r**_2_σ_2_, ..., **r**_*N*_σ_*N*_) of *N* electrons of spins σ_*i*_ and positions **r**_*i*_, we can write Ψ(**X**) as
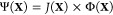
1where Φ(**X**) takes into account the fermionic nature of the electrons, while *J*(**X**) is the JF: an exponential modulation of the WF that substantially improves the electronic correlation description for all types of WFs studied here. The fermionic term of the WF, dubbed as Φ(**X**) in eq 1, is the most important part, directly encoding the behavior of the electrons while imposing the antisymmetrization under particle exchange. In the following section, we will describe the basis set used, the definition of the AGPs, AGPu, and AGP after a brief introduction to the SD. Finally we will discuss the JF correlator.

### 2.1 Basis Set

We expand our ansatz in an atom-centered basis set of Gaussian orbitals for the calculation of the JF and a hybrid basis set for the fermionic part of the WF, as it will be discussed below. The Gaussian orbitals basis set is indicated as {ϕ_*I*,ν_(**r**)}, with each element being the νth orbital centered on the *I*th atom at the position **R**_*I*_. The elements in the basis set have the form
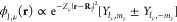
2where *Z*_ν_ is a numerical coefficient that describes how diffused the atomic orbital is around the atom, while *Y*_*l*_ν_,*m*_ν__ is the spherical harmonic function with angular quantum numbers *l*_ν_ and *m*_ν_, corresponding to the orbital type ν which is always assumed to be real. This basis set has been used without further contractions for the description of the JF. Instead, for the fermionic part of our WF, we have used hybrid atomic orbitals (HOs)^26,27^ to expand them over a richer set of Gaussian orbitals and, by means of the contraction, considering only an affordable number of variational parameters. The HOs, indeed, are obtained as linear combinations of all the elements of the Gaussian basis set used for a given atom, labeled by *I*
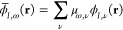
3

The above hybrid orbitals allow us to take into account the modification of the standard Slater orbitals corresponding to isolated atoms, to the case when they are instead placed in a complex environment. Therefore, we have chosen to use a number of hybrid orbitals equal to the single particle ones occupied in the absence of electron–electron interactions and including also all the ones corresponding to the same shell of degenerate one particle levels. The corresponding orbitals are the ones that should physically play a role in the considered electronic systems. Hence, in all of the first row molecules, we have considered the full hybridization of five atomic orbitals, coming from two s-wave and three p-wave ones, that can be corrected by several components with much higher angular momenta. This is because the full spherical symmetry is no longer satisfied even in a simple homonuclear molecule. For the sake of compactness, we indicate here all basis elements as {ϕ_*k*_(**r**)} combining the indices ω and *I*, and *I* and ν in a single index *k* for a lighter notation. Every time we refer to the AGPs, AGPu, and AGP, it is meant to be a basis of HOs.

The exponents *Z*_ν_ have been chosen from the ccpVDZ or ccpVTZ basis set according to this criterium: the contraction are removed and all exponents with *Z*_ν_ > 150 a.u^–2^. are eliminated. This is possible because contracted orbitals containing very large exponents are necessary only with a pure Gaussian basis in order to satisfy the electron-ion cusp conditions. They are instead appropriately considered by the one-body term of our JF, as described in Section 2.4. The exponents chosen are then further optimized at molecular equilibrium distance and kept fixed in the corresponding atomic calculation (where the optimization of the exponents has an almost negligible effect) and the dispersion energy curves.

### 2.2 Slater Determinant

Here, we will provide a preparation description of the SD that is important both for the initialization of the pairing function and for comparing our results with the existing literature.^17^ From theoretical and computational point of view, the simplest fermionic WF is the SD, called Jastrow SD (JSD) in the presence of a JF. The SD is built from the vacuum state by populating a number of orthogonal single particle molecular orbitals (MOs) equal to the number of electrons in the system. Henceforth, we omit the spin indices, by assuming that each spin component corresponds to a different SD. In our basis, the MO are in the form
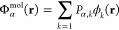
4

The MOs can be obtained directly from a density functional theory (DFT) or Hartree–Fock calculation, but they can also be further optimized with VMC.^43^ It is well known that the antisymmetric product of these MO leads to the determinant of the matrix in which every MO is evaluated for each electron position
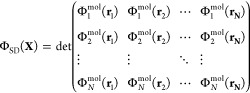
5

For weakly correlated systems, the JSD can often give reasonably good results with an affordable computational cost and a limited number of variational parameters. It is also a common choice to use a linear combination of SDs to improve the description of the WF, with *ansatzs* that take different names depending on the type and number of SDs considered. In this paper, we will compare directly the results of our WFs to the ones obtained with one of the most successful multideterminant WFs, the full valence complete active space (FVCAS) WF.

### 2.3 Pairing Function

The use of the pairing function in correlated WFs allows an electronic description that goes beyond the single particle picture of the SD. The building block of this WF has the following general form
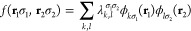
6where all the elements of the matrix λ represent most of the WF variational parameters. They depend on the orbitals considered and on the spin σ_1_, σ_2_ of the so called geminal function *f*. In principle, when we break the spin symmetry, the basis sets used for ↑ and ↓ electrons can be different, otherwise the chosen basis does not depend on the spin component. In order to set up a consistent many-body WF starting from the geminal, several choices are possible depending on the criteria adopted for the definition of the geminal. To highlight the different possibilities, we can recast eq 6 in a way in which the spin dependency is more explicit
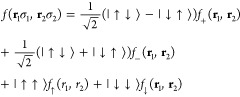
7where
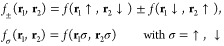
8In order to satisfy the Pauli principle, we have *f*_±_(**r**_1_,**r**_2_) = ±*f*_±_(**r**_2_,**r**_1_) and *f*_σ_(**r**_1_,**r**_2_) = −*f*_σ_(**r**_2_,**r**_1_) for σ = ↑, ↓. Our WF is then obtained by antisymmetrizing the product over all electron pairs considered that, by definition, occupy the same pairing function. For simplicity, we will enumerate the spin up electrons from 1 to *N*_↑_ and the spin down ones from *N*_↑_ + 1 to *N*.

As suggested by the name AGP, our goal is to define a WF that is literally the antisymmetrized product of the geminals and the unpaired orbitals (if present), namely
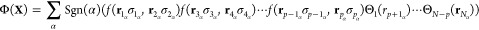
9where α is one of the possible way of distributing the *N* electrons between the *p*/2 pairs and the *N* – *p* unpaired orbitals Θ, and Sgn(α) is the sign of the corresponding permutation of the particles that is required to ensure the fermionic behavior. In particular, different choices of the pairing function, obtained by excluding one or more terms in the eq 8, lead to different ways to compute eq 9. These choices also impact quantitatively and qualitatively on the kind of physics that we can describe by means of this type of WF. Therefore, we will distinguish among three distinct cases: if we consider only the singlet term in eq 8 we obtain the AGPs, if we include the singlet and the *S*_*z*_ = 0 triplet term we have the AGPu, while the most general case is just the definition adopted here for the AGP.

#### 2.3.1 AGPs

Let us consider for the moment the unpolarized case *N*_↑_ = *N*_↓_, the extension to the polarized cases will be straightforward and will be discussed later on. When no triplet correlations are allowed, we build our WFs using only singlet pairs, and the pairing function in eq 7 contains only the symmetric element *f*_+_
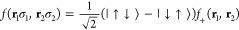
10In this case, we project a perfect singlet that we denote as AGPs. The λ matrix elements in eq 10 are nonzero only for σ_1_ ≠ σ_2_, and they are symmetric for spin exchange. In order to calculate the AGPs, we can write all the possible combinations of pairs of opposite spin electrons in a matrix defined as
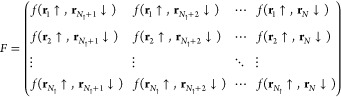
11In this way, each row of the matrix corresponds to an electron of spin ↑ and each column an electron of spin ↓. The definition of the matrix *F* in this form is convenient because it allows the antisymmetrization requested by the eq 9 in a simple and efficient way. Indeed, it can be demonstrated^26^ that the correct antisymmetrization of the pairs considered in this case is given by
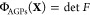
12This is somehow intuitive because we want to sum all the possible products of *N*/2 matrix elements of *F*, where in all these factors, a column element or a row element is present only once, exhausting all the possible configurations of the system considered with an appropriate ± sign that, in this case, is just given by the one corresponding to the determinant of *F*.

When the system is polarized and *N*_↑_ ≠ *N*_↓_, we cannot build the solution using only the singlet terms because the matrix *F* written as in eq 11 is a rectangular matrix and its determinant cannot be computed. Supposing for simplicity that *N*_↑_ > *N*_↓_, in this case, we have to add a number *N*_↑_ – *N*_↓_ of unpaired spin-up MOs {Θ_*i*_(**r**)} not only for fulfilling the polarization required but also, most importantly, to turn the matrix *F* to a perfectly defined square matrix
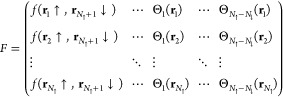
13Also in this case, a consistent antisymmetric WF can be again calculated as the determinant^26^ of the matrix *F* exactly in the same way as the singlet pairing in eq 12.

#### 2.3.2 AGPu

For the AGPu, only the parallel spin term of the triplet component are omitted. This means that the spin symmetry is broken, and a magnetic order parameter can be directed along the *z*-quantization axis. This WF is called the broken symmetry AGP (AGPu), and the difference from the previous AGPs is the presence of the antisymmetric *f*_–_ component in the definition of the pairing function in eq 7, that for this case is
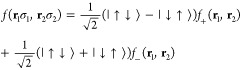
14

In order to define this pairing function, we break the spin symmetry in the opposite electron spin case with σ_1_ ≠ σ_2_, by keeping equal to zero the σ_1_ = σ_2_ components of eq 6. With exactly the same procedure used in the case of the AGPs, depending on the polarization, we can build the same matrix *F* of eqs 11 or 13 that is no longer symmetric now. Even in this case, the correct antisymmetrized sum of these pairs is given by the determinant.^26^ Thus, analogously to eq 12, we obtain
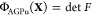
15that implements the simplest broken symmetry *ansatz* based on the pairing function.

#### 2.3.3 AGP

The AGP (also known in the literature as Pfaffian WF^23^) is in our opinion the most important pairing function, being the most general one and encoding new variational freedoms into the AGPs and the AGPu. We will show that it represents the most powerful description of the chemical bond within the paradigm developed in this work. This WF represents also the most general mean-field state, that is, the GS of a mean-field Hamiltonian containing BCS anomalous terms projected on a given number *N* of particles and total spin projection  along the *z*-quantization axis. In this case, the definition of the pairing function is exactly the one in eq 7, containing all terms including the parallel spin terms of the triplet. This means that now, when we build the AGP, we have to include also the parallel spin electron pairs in the WF. In this way, the AGP can also describe a magnetic order parameter in any direction of the space, and thus, it is also possible to rotate the spin component of the WF in any direction. This will allow us to break the symmetry along the spin-quantization axis and then rotate it. As we will explain later, this plays a crucial role when we use this WF in combination with our JF because it allows us to preserve the total *S*_*z*_ of the molecules and include spin fluctuations.

Of course, we cannot create a WF using only pairs if the number of electrons in the system is odd, so, for the moment, let us assume *N* is even. The extension to the odd number of electrons is trivial and will be discussed immediately after. We will dub as *W* the matrix containing all possible pairs
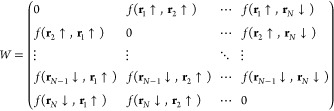
16where the matrix is antisymmetric for the fermionic commutation rules, and thus, the elements of the diagonal are set to zero. We can recast the *W* highlighting its different spin sectors as
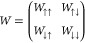
17where *W*_↑↑_ and *W*_↓↓_ are respectively *N*_↑_ × *N*_↑_ and *N*_↓_ × *N*_↓_ antisymmetric matrices that take into account the parallel spin terms of the triplet, while *W*_↑↓_ is a *N*_↑_ × *N*_↓_ matrix such that *W*_↑↓_ = −*W*_↓↑_^T^, describing the remaining triplet and singlet contributions. In the case of AGPs and AGPu, we can also build a similar matrix where the matrices *W*_↑↑_ and *W*_↓↓_ are identically zero.

Analogously to the case of the AGPs and AGPu, we have to identify a way to calculate the antisymmetric product of all pairs considered. In this case, it is easy to identify the antisymmetrization procedure defined in eq 9 as the Pfaffian of the matrix *W*. After introducing this algebraic operation, the reason will be straightforward to the reader.

The Pfaffian is an algebraic operation acting on antisymmetric square matrices with an even number of rows and columns. Being *N* even, the matrix *W* satisfies these hypothesis. The usual definition of the Pfaffian requires the introduction of the concept of partition of the matrix *W*
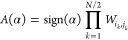
18where all *i*_*k*_ and *j*_*k*_ are different, *i*_*k*_ < *j*_*k*_ for each *k* and *i*_1_ < *i*_2_ < ... < *i*_*N*_. The sign(α) is given by the permutation that orders the vector of the indices {*i*_1_, *j*_1_, *i*_2_, *j*_2_, ..., *i*_*M*_, *j*_*M*_}. In this way, all indices are considered only once. The Pfaffian is then defined as
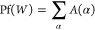
19where the sum over α is extended over all possible partitions. However, an alternative definition^44^ of the Pfaffian can better clarify the correspondence to the eq 9. It can indeed be defined alternatively as
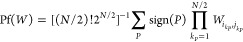
20where *P* now represents a generic permutation of the possible row and column indices of the matrix without any constraints, and the sign(*P*) is the parity of the permutation. In this definition, it is easy to recognize the antisymmetrized sum corresponding to the eq 9. Let us introduce now a further property of the Pfaffian that will be useful in the following section. We will indicate with 0 a *m* × *m* matrix containing only 0 and B a generic *m* × *m* matrix, we have
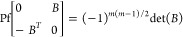
21

For odd number of electrons, it is necessary to use a spin-dependent unpaired orbital Θ_σ_(**r**) so that we can accommodate the remaining electron that is not considered by the product of the pairs. The unpaired orbital introduces a supplementary row and column to the matrix *W*. Being Θ_↑_ = (Θ_↑_(**r**_1_), Θ_↑_(**r**_2_), ..., Θ_↑_(**r**_*N*↑_)) the vector containing the values of the unpaired orbital Θ_↑_ at the ↑ electron positions and Θ_↓_ = (Θ_↓_(**r**_*N*_↑_+1_), Θ_↓_(**r**_*N*↑+2_), ..., Θ_↓_(**r**_*N*_)) the one calculated for the ↓ electron ones, we modify the matrix in eq 17 as
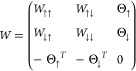
22Also in this case, the permutation sum implied by the Pfaffian leads to the correct antisymmetrization required from eq 9. The matrix *W* satisfies the hypothesis of the calculation having an even leading matrix dimension *N̅* = *N* + 1. We can further notice that no assumption has been made on the polarization of the system, and so no unpaired orbital is required except for a single one in the case of odd *N*.

It is however possible in principle to introduce further pairs of unpaired orbitals, if, for example, we want to describe AGPs or AGPu with a full AGP WF. We define Θ_*i*σ_(**r**) as the set of the considered *m* unpaired orbitals and Θ_*i*_↑__ = (Θ_*i*,↑_(**r**_1_), Θ_*i*,↑_(**r**_2_), ..., Θ_*i*,↑_(**r**_*N*_↑__)) the vector containing the values of the unpaired orbital Θ_*i*,↑_ for the ↑ electron positions and Θ_*i*_↓__ = (Θ_*i*,↓_(**r**_*N*_↑_+1_), Θ_*i*,↓_(**r**_*N*_↑_+2_), ..., Θ_*i*,↓_(**r**_*N*_)) the one calculated for the ↓ electron ones. We can modify the matrix in eq 17 as
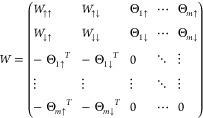
23that is a *N̅* × *N̅* matrix where *N̅* = *N* + *m*. We can again antisymmetrize this product using the definition of the Pfaffian provided in eq 20. A careful reader could have noticed that, by applying the Pfaffian definition, we are antisymmetrizing not only over the electron indices but also over the orbital indices of the unpaired orbitals. This antisymmetrization, however, contains the one over the physical electrons, and leads therefore to a physically allowed electronic WF.

Moreover, we can notice that, by using the previous definition, we can identify the AGPs and the AGPu as sub-cases of the general AGP. Indeed, by using the expressions of the pairing function and the unpaired orbitals of the AGPs and AGPu, we obtain *W*_↑↑_ = 0, *W*_↓↓_ = 0, Θ_*i*_↓__ = 0, and *N̅* = 2*N*_↑_. By merging eqs 13 and 17, we can define
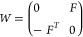
24and this means that, by applying eq 21, we immediately obtain
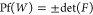
25where the sign only depends on the number of electrons and is constant, thus irrelevant. This shows in a straightforward way that the AGPs and AGPu defined in the previous subsection are nothing but particular cases of the most general AGP.

### 2.4 Jastrow Factor

Within QMC, it is easy to improve the quality of the WF by multiplying the WF with an exponential JF. This last one enriches the description of the GS by encoding explicitly the electronic correlation, while speeding up the convergence to the complete basis set limit.^7^ Indeed, with an appropriate choice, the JF can satisfy exactly the electron–electron and electron-ion cusp conditions of the many-body WF, consequences of the Coulomb 1/*r* singularity at a short distance. In this paper, we introduce a new kind of JF that contains a richer dependence on the spin and that plays a fundamental role when used in combination with the AGP WF. The JF is defined as
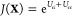
26where *U*_*ei*_ is a single body term that deals explicitly with the electron–ion interaction and *U*_*ee*_ is a many-body term that properly accounts for the electronic correlation. The single body term is
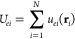
27with *u*_*ei*_ being
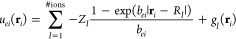
28In eq 28, *Z*_*I*_ is the atomic number of the atom *I* and *b*_*ei*_ is a variational parameter, while *g*_*I*_(**r**_*i*_) encodes the most general nonhomogeneous electron–ion one-body term, that is, depending explicitly on all nuclear and electron coordinates and not only on their relative distances, that is defined as
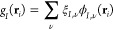
29where the summation is extended over all Gaussian orbitals in the JF basis set centered on the *I*th atom. The electron–electron term instead is written as
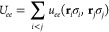
30where the sum is extended over the pairs of different electrons and where
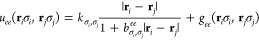
31with the 2 × 2 matrix  described by one  or two variational parameters for σ_*i*_ = σ_*j*_ when *k*_σ_*i*_,σ_*j*__ = 1/4 and  for σ_*i*_ ≠ σ_*j*_ when *k*_σ_*i*_,σ_*j*__ = 1/2 and . The conventional expression for the JF can be obtained by removing all spin dependencies in the previous expressions and considering only the variational parameters corresponding to the opposite spin case *k*_σ_*i*_,σ_*j*__ = 1/2 and .

In our expression, the first term in eq 31 deals explicitly with the electron–electron cusp conditions, the second term in eq 31 instead is a bosonic pairing function in the form
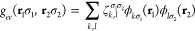
32with the elements of the matrix ζ defining further variational parameters. Notice that both *g*_*I*_ and *g*_*ee*_ do not affect the cusp conditions because they are expanded over cuspless Gaussian orbitals. The *g*_*ee*_ term has the same form of eq 6, but since the fermionic behavior is already encoded in the fermionic part of the WF, this term is symmetric under particle exchange. The use of a pairing function in the JF enriches the description of the charge and spin correlations of the system, noticeably improving the quality of the global WF. It is a common practice to adopt a simplified or even absent spin dependency in the function *u* of eq 31. This is often accurate for systems where the magnetic properties are not relevant. We will refer to it below with the prefix Js in the WF, in contrast with the prefix J used for the full spin-dependent JF.

A perfect singlet remains as such after the multiplication of a spin-independent Jastrow, and so our spin-dependent JF is not appropriate if we do not want to break the spin symmetry. It is, instead, necessary if we want to recover, at least approximately, the singlet from a spin contaminated broken symmetry ansatz. A general spin-dependent *u*, as defined in eq 31, is therefore of fundamental importance for the AGPu or the AGP ansatzs.

Let us start with a simple example. We consider two atoms with opposite spins and break the spin symmetry by orienting the spins of the atoms along the *z*-quantization axis. In this case, the JF is not able to change the classical antiferromagnetic spin state because it acts as an irrelevant constant when applied to it. It is instead more physical to orient the spin moment of the atoms in a direction perpendicular to the quantization axis chosen for the JF. In this way, the JF can act on the electrons and the spins while the magnetic moment is free to fluctuate and recover its genuine quantum character. As previously mentioned, with the AGP it is possible to rotate the spin of the WF in every direction and orient the magnetic moment in any direction of the space. This works particularly well in combination with our JF that can suppress the unfavored triplet configurations with parallel spins generated by the rotation. This optimal spin orientation of the atoms, that is perpendicular to the JF one, is rigorously valid within the well-known spin-wave theory of a quantum antiferromagnet.^34^ In this case, the JF defined with a spin-quantization axis perpendicular to the magnetic moment of the atoms allows the description of the quantum fluctuations and the corresponding zero point energy (ZPE), even for a finite (as is our case) number of atoms.^34^

### 2.5 Procedure

The first step to calculate and optimize our WF is to identify a reasonable starting point. We chose to start from a DFT calculation because of its flexibility. We have used LDA calculations for spin symmetric systems, while for the ones with opposite spin antiferromagnetic moments we have broken the symmetry with a LSDA calculation, by adopting an appropriate initialization of the WF. The SD obtained from a DFT calculation is mapped without loss of information into AGPs or AGPu and then in a second analogous step, we convert the AGPs and AGPu into a full AGP.

For the first conversion, let us consider eq 6. If we compute it in the basis set of the MOs obtained from the DFT, we have
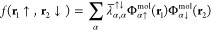
33namely only the diagonal terms in the matrix λ̅ are present. Moreover, we can also remove the spin dependency if there is no symmetry breaking. For the polarized case, the unpaired orbitals are the last occupied MOs. By substituting the definition of the MOs with their expansion over a localized atomic basis set, as given in eq 4, we can recast the above equation exactly in the same form shown in eq 6 with a matrix λ
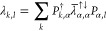
34

When we convert AGPs or AGPu into an AGP WF, we already have an initialization for the sectors of the pairs with different spins *W*_↑↓_ and *W*_↓↑_ that can be obtained directly from the AGPs or AGPu pairing functions. The main challenge is to find a reasonable initialization for the two sectors *W*_↓↓_ and *W*_↑↑_ that are not described by the AGPs or AGPu.

There are two different procedures that we can follow: the first one is used for polarized systems, and the second one instead is preferred in the case of broken spin symmetry and in the presence of antiferromagnetism, namely, molecules well described by opposite atomic magnetic moments. If there is no antiferromagnetism and the polarization is such that |*S*_tot_^*z*^| < 1, the *W*_↓↓_ and *W*_↑↑_ are instead identically zero. This holds true not only for *S*_tot_^*z*^ = 0 but also for *S*_tot_^*z*^ = ±1/2, where the single unpaired MO used in eq 22 acquires also a spin dependency, not present in the AGPs and AGPu cases.

Obviously the atoms, and also the O_2_ molecule, do not have antiferromagnetism, but, on the other side, they have a net polarization. We can build the *W*_↑↑_ block of the matrix using the two unpaired orbitals Θ_1_ and Θ_2_ for the definition of the parallel spin matrices of the AGPs or AGPu in the following way
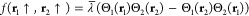
35where the presence of the minus sign guarantees the pairing function to be antisymmetric under particle exchange, while the λ̅ is an arbitrary scaling factor that has no influence on the final value of the WF. Once we map the unpaired orbitals in the desired basis set, we obtain the variational parameters of the matrix λ for the ↑↑ sector.

In the presence of opposite atomic magnetic moments, it is possible to rotate the spin component of the pairing function to initialize the *W*_↓↓_ and *W*_↑↑_ sectors. As we mentioned earlier, a further effect of this operation is to direct the atomic magnetic moments in a direction perpendicular to the spin-quantization axis. It is worth mentioning that, within our method, the spin orientation with respect to the molecular axis is irrelevant because in a nonrelativistic Hamiltonian, the spin–orbit coupling is not present. In this case, we have chosen to work with the atomic magnetic moments perpendicular to the *z*—axis, hence we applied a rotation of π/2 around the *ŷ* direction. This operation maps
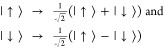
36If we apply this transformation to the pairing function from eq 14, we obtain
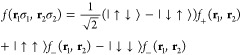
37

This transformation provides a meaningful initialization to our AGP WF that now has to be optimized to reach the best possible description of the GS. Indeed, within VMC, it is only thanks to the optimization that we can improve the description of the GS. So far, we have only converted the DFT WF from one *ansatz* to another, but the key for the success of this procedure is the optimization of all possible variational parameters. It is indeed crucial to optimize not only the ones corresponding to the matrix λ and the JF parameters but also the coefficients of the hybrid orbitals μ and the exponents of the Gaussian basis set *Z*_ν_. This is realized computationally in a very efficient way using a coding technique called adjoint algorithmic differentiation^45^ that allows calculations of total energy derivatives with respect to all variational parameters involved in a given algorithm that computes only the energy. This is remarkably done by paying a very small slowing down of a factor ≈2–3 with respect to the latter algorithm. We have also used a state of the art optimization scheme^46,47^ for a correct search of the energy minimum. Remarkably, even when there is some possible dependency among the many variational parameters considered in our ansatz, the stochastic reconfiguration technique remains stable and efficient, thanks to an appropriate regularization of the stochastic matrix *S*.^10^ Once we calculate the variational minimum, the best description of the GS is then obtained with the DMC calculation.

Even considering that the number of variational parameters involved in the calculation may be quite large, the optimization has a very small impact over the total computational cost, that is indeed mostly given by the DMC for all cases reported in this work. In Table 1, we compare the computational cost of the DMC calculations for the different WFs considered. We notice that the JAGP is even less expensive than the JSD and JsAGPs WFs. The JAGP and JAGPu are so efficient because, in this case, the variance of the energy is considerably smaller, as we can see from Table 1. This implies that JAGP and JAGPu require a smaller number of DMC iterations to reach the desired accuracy because they have lower variance compared with the JsAGPs and the JSD, thanks to the spin-dependent JF.

**Table 1:** DMC Computational Time Required to Obtain an Accuracy of 0.1 mH and Energy Variance on the Oxygen Dimer with an Intel Xeon Architecture Using a Recent LRDMC Algorithm^48^ With a Lattice Spacing Equal to 0.05 bohr, the Smallest Used in This Work^a^

WF	CPU time	variance [*H*^2^]
JSD	2806	2.909
JsAGPs	2526	2.819
JAGPu	14,523	2.455
JAGP	1857.79	2.125

a In these systems, the cost for doing about 10,000 iterations for the VMC optimization of our WFs is less than 30 h. The CPU time reported in the table corresponds to the total one (time spent by a single core times the number of cores) for obtaining the required accuracy, for example, with 256 cores parallel computation, the JAGP calculation can be obtained with about 7 h of walltime.

For large number *N* of electrons, the DMC calculation should scale as *N*^4^ for fixed total energy accuracy, and the main question, that we have not studied here, is whether the optimization remains computationally negligible because the number of variational parameter scales as *N*^2^. In this respect, we have experienced that an optimization technique performed with a slow but very stable method, that is, with a large number of “cheap” optimization steps, each one determined by a relatively small number of samples (even much smaller than the number of parameters) is very promising for future large *N* applications.

Finally we introduced a technique to deal with particularly unstable AGP WFs. Indeed, it is possible, after a very large number of optimization steps (>10,000), that some eigenvalues of the matrix λ become too small as compared with the largest reference eigenvalue. This creates some instabilities in the inversion of the matrix *W* required for the QMC fast updates. For this reason, by an appropriate use of the PFAPACK library,^49^ we identified a procedure to map the diagonalization of a full skew-symmetric matrix λ to the one corresponding to a real tridiagonal symmetric matrix. After this mapping, we can use the most powerful and stable LAPACK routines for diagonalization. Indeed, most linear algebra packages cannot deal with antisymmetric matrices, and a general diagonalization tool was not available for this case. The introduction of this procedure, described in detail in the Appendix, allows us to describe the matrix λ in terms of eigenvalues and orthogonal orbitals playing the role of eigenvectors of an antisymmetric matrix. We will refer to them in the Appendix as MOs because it may be considered their formal definition, within the formulation introduced in this work. With this meaningful decomposition, we can finally regularize the matrix λ by replacing the too small eigenvalues with reasonable lower bounds and continue, if necessary, with the optimization of the variational parameters.

### 2.6 *S*^2^ Operator

The basic concept of QMC relies on the real space configurations sampling of a general electronic system. All observables can be indeed calculated in the basis where the electron positions and their spins are defined. In particular, for the systems considered, it is interesting to estimate the spin observables in order to understand their magnetic properties and the quality of the corresponding WFs. If during the simulation the value of *S*_*z*_ is fixed, when we break the symmetry the value of the *S*^2^ is instead the result of the interplay between the JF and the AGP or the AGPu. The efficient computation of the expectation value of the *S*^2^ operator has already been described in ref (50) for the JsAGPu and will be shown now for the JAGP.

Here, we show how to evaluate *S*^2^ in a region of the space with a fast and computationally cheap approach based on the fast update algebra of the AGP and the spin-dependent JF. Let us consider the expectation value of the *S*^2^ operator over a generic WF Ψ by direct application of its definition. Here, we use the completeness of the spatial configurations
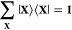
38where the summation symbol implies a 3*N*-multidimensional integral over the electron coordinates. Assuming a fixed polarization, we can write the explicit expression of the total spin square as
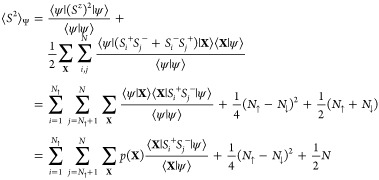
39where the operators *S*_*i*_ in the above equation act on the spin component corresponding to the electron position **r**_*i*_ of the configuration **X**. We can notice that
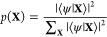
40and that, by using QMC sampling, we generate configurations according to the probability density *p*(**X**). Thus, we can evaluate the above multidimensional integral by directly sampling the estimator *S*^2^(**X**) that multiplies *p*(**X**) in eq 39, as follows
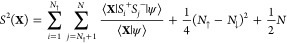
41

The content of the former equation can be evaluated efficiently as we will explain in the following section. Indeed, the application of the operator *S*_*i*_^+^*S*_*j*_^–^ to the configuration **X** generates only a configuration **X**_*ij*_ = {(***r***_1_↑), ..., (***r***_*i*_↓), ..., (***r***_*j*_↑), ..., (***r***_*N*_↓)}. Considering **X** our sampled configuration and using the previously given definition of **X**_*ij*_, we can recast eq 41 as
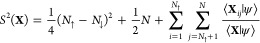
42The only hard challenge of eq 42 is the calculation of the *N*_↑_× *N*_↓_ ratios
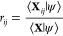
43for *i* = 1, 2, ..., *N*_↑_ and *j* = *N*_↑_ + 1, *N*_↑_ + 2, ..., *N*, that in our case read
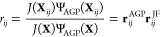
44The configurations **X** and **X**_*ij*_ differ for a spin flip of the electrons *i* and *j*, but we can also consider **X**_*ij*_ as the configuration in which the electron *i* evolved to the position previously occupied by *j* and vice versa. We can then calculate the ratios in eq 44 using a fast algebra to update two positions for the AGP and for the JF with a direct evaluation based on the Sherman–Morrison algebra and some simple manipulations, as discussed in detail later on.

It is also possible to calculate the value *S*^2^(Λ) of the *S*^2^ operator in a sub-region of the space Λ. For this quantity, we can obtain the similar expression to eq 42
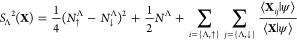
45where *N*_σ_^Λ^ (σ = ↑, ↓) is the number of σ-electrons in the region Λ, *N*^Λ^ = *N*_↑_^Λ^ + *N*_↓_^Λ^. The summation symbol over *i* ∈ {Λ, σ} indicates the sum for all σ-electron whose coordinate is in the region Λ. Therefore, we can use the same method as described below.

#### 2.6.1 AGP Contribution

To calculate the AGP contribution to *r*_*ij*_, we were able to find a slim and fast algebra making an extensive use of the Pfaffian properties.^51^ It was fundamental to find an efficient algebra to calculate the whole matrix of the ratios *r* with a computational cost that is *O*(*N*^3^), by using mostly BLAS3 operations, thus avoiding that this computation could become the bottleneck of the whole procedure. In this way, we could ensure the evaluation cost of *S*^2^ to be comparable with the one of a typical QMC cycle over all *N* electrons that is at most *O*(*N*^3^). Before describing the fast updating rules for the position of two electrons with a single move, we need to introduce some quantities fundamental for the calculation.

Let us denote *W*^–1^ as the inverse of *W*. This inverse *W*^–1^ can be computed from scratch for each configuration used to sample the spin square. The electron coordinates **r**_*i*_ are given for *i* = 1, ..., *N*, but since the corresponding spin can change with respect to the original choice (↑ for *i* ≤ *N*_↑_, and ↓ for *i* > *N*_↑_) because of the spin flips mentioned in the previous subsection, we will consider explicitly the values of the spin here.

We then define the matrix θ as
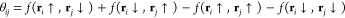
46For the spin ↑ electrons, we can define the vectors
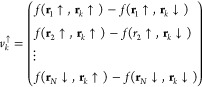
47while for the spin ↓, we have instead
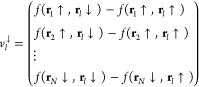
48We can use these vectors to build the *N* × *N* matrix
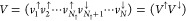
49that allows us to define
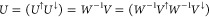
50and finally
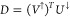
51

Now, we have all ingredients that we need for our fast updating algebra, and upon application of Sherman–Morrison algebra, we arrive at the ratio
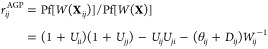
52We can notice that the preliminary calculation of the auxiliary matrices θ, *V*, *U*, and *D*, including the inversion of *W*, amounts to a total of *O*(*N*^3^) operations, while the calculation of the ratios is *O*(*N*^2^) once the matrices have been computed.

#### 2.6.2 JF Contribution

In the JF that we introduced in the previous section, only the two-body term of eq 30 has a spin dependency, and thus, only this part contributes to the ratio. By simple substitution, it is easy to prove that
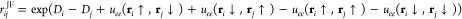
53where we have defined
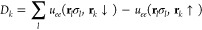
54The whole operation has a *O*(*N*^2^) computational cost and so does not limit the calculation in terms of performances.

## 3 Results and Discussion

We apply this new approach for two types of systems: the first row high spin atoms (carbon, nitrogen, and oxygen) and their diatomic molecules and the benzene molecule. The first ones still represent useful benchmarks for the quantum chemistry approach, and a reasonable description of their properties and binding energies requires very expensive multireference methods. It is therefore very interesting to test our approach to find whether we are able to obtain a good description with a single Pfaffian *ansatz*. Benzene molecule on the other side is the most famous and important example of the RVB theory, so it represents a fundamental benchmark test for a method inspired by this theory. Here, we compare our results with exact available solutions, JSD WFs and with the JFVCAS multideterminant expansions for QMC. We will also show that our WF satisfies the size consistency both at VMC and DMC levels, a primary requirement if we want to use this approach for more challenging chemical studies.

### 3.1 Carbon

Carbon dimer is probably the most interesting example discussed in this study. A full understanding of the behavior of the carbon–carbon interaction is still missing, and the bond order of this molecule is still under debate.^52^ The role of the spin fluctuations in this molecule has already been discussed,^53^ but we believe that it is very instructive and represents the most important achievement of the JAGP WF. Indeed, it is only thanks to the spin fluctuations that we can have a correct description of its dimer bond.

**Figure 1:**
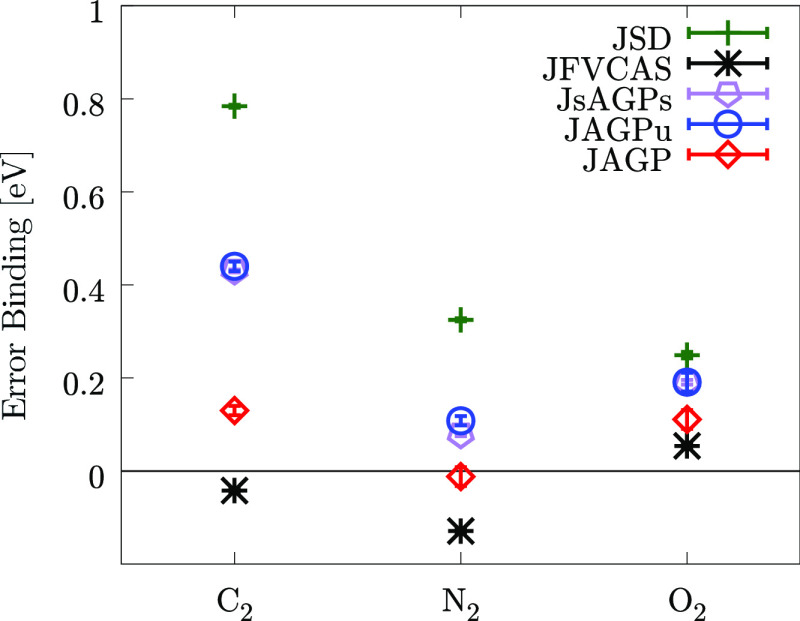
Comparison of the different DMC energies for different WFs. The results are shown for the three dimers described in this paper. The JFVCAS and the JSD results are taken from the literature.^17^

The carbon atoms have spin triplet electronic configurations, and their mutual interaction leads to a singlet molecule. As we can see from Figure 1 and Table 3, the JAGP not only improves the results of the JSD WF but remarkably also the description given by the JsAGPs and JAGPu. The huge difference between the multideterminant expansion JFVCAS and the JSD binding energies helps to quantify the effect of the multideterminantal nature of this molecule, and what makes this even more surprising is that the quality of the results obtained with a single JAGP WF, with a computational cost comparable to a SD, is already very close to the exact value.

**Table 2:** Spin Measures with Different WFs for the Carbon Atom and Dimer at VMC Level

	*S*^2^		2μ_B_
	atom	molecule	moment∥*z*
JsAGPs	2.00	0.00	0.0005(4)
JAGPu	2.00534(3)	0.1743(5)	0.5833(4)
JsAGP	2.00418(5)	0.2880(4)	0.7194(4)
JAGP	2.00542(1)	0.0327(1)	0.0013(5)
exact	2.00	0.00	

**Table 3:** Carbon Energies^a^

	**carbon**		
	atom	molecule	binding
source	energy [H]	energy [H]	energy [eV]
JSD	–37.81705(6)^b^	–75.8088(5)^b^	4.75(1)^b^
JFVCAS	–37.82607(5)^b^	–75.8862(2)^b^	6.369(6)^b^
JsAGPs	–37.8243(1)	–75.8611(2)	5.78(1)
JAGPu	–37.8263(1)	–75.8706(2)	5.93(1)
JAGP	–37.827965(3)	–75.88650(4)	6.274(3)
JSD (DMC)	–37.82966(4)^b^	–75.8672(1)^b^	5.656(3)^b^
JFVCAS (DMC)	–37.83620(1)^b^	–75.9106(1)^b^	6.482(3)^b^
JsAGPs (DMC)	–37.8364(1)	–75.8938(2)	6.01(1)
JAGPu (DMC)	–37.8364(1)	–75.8935(2)	6.00(1)
JAGP (DMC)	–37.8363(1)	–75.9045(2)	6.31(1)
estimated exact	–37.8450^c^	–75.9265^d^	6.44(2)^d^^,^^e^

a The JsAGPs, JAGPu, and JAGP results are calculated with an optimized ccpVTZ basis set.

b Reference (17).

c Reference (54).

d Reference (55).

e A more recent estimate yields 6.39 eV (Cyrus Umrigar, private communication).

**Figure 2:**
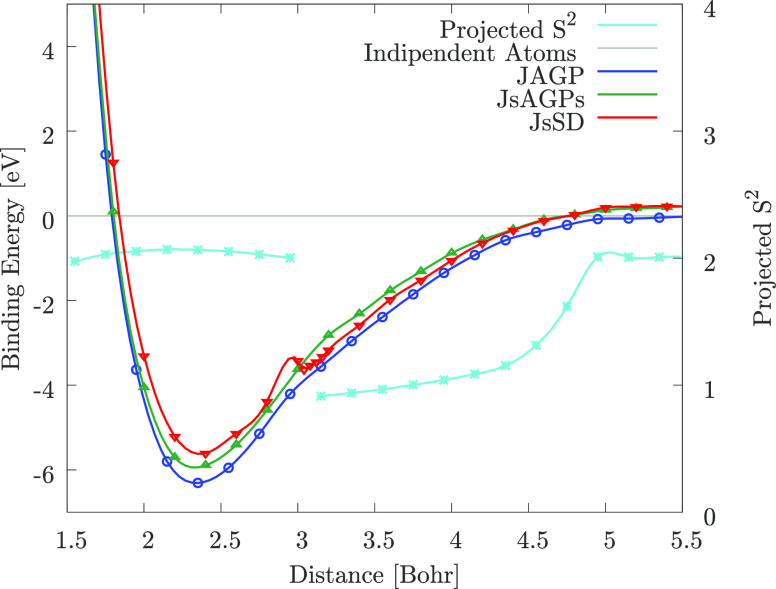
DMC energy dispersion of the carbon dimer: only the JAGP allows the system to be size consistent at large distance, which means that it is able to recover the energy and the expectation value of the *S*^2^ operator of two isolated atoms at bond distance; however, the carbon atoms maintain a large value of *S*^2^. The sharp change of the projected *S*^2^ value at around 3 a.u. is probably due to an avoided crossing of two energy levels belonging to the same irreducible representation, in agreement with DMRG.^59^ Within LSDA, this effect is reproduced by a discontinuous change in the occupation of the π orbitals in the corresponding SD. Lines are guides to the eye.

As already mentioned before, the explanation for the impressive improvement of the binding energy from JsAGPs and JAGPu to JAGP resides on the description of the strong spin fluctuations in this molecule. The JAGP gives a very accurate picture of its magnetic properties as we can see from Table 2, giving results very close to *S*^2^ = 2 for the atom and *S*^2^ = 0 for the molecule. Conversely, by using the JsAGP (the AGP without spin-dependent JF) and the JAGPu, we cannot recover the singlet from the broken symmetry initialization. Interestingly, as expected, the molecule does not have any magnetic moment on the *z*-direction because it is an almost perfect singlet. The atomic spins, localized around each atom, point in opposite directions in order to form the singlet molecular state. Because there is no magnetic moment along *z*, we can measure its magnetic moment only by separately evaluating the *S*^2^ in the two semi-infinite regions, each one containing a single atom, separated by a plane perpendicular to the molecular axis and at the same distance from the two atoms. In Figure 2, we show that, even at bond distance, there is a very strong magnetic moment around the atoms and, in this way, we can explain the strong effect of the ZPE of the spin fluctuations described by the JAGP.

Moreover, Figure 2 shows that only with the JAGP WF, we have a size consistent solution with the molecule that recovers the energy of two independent atoms at large distance. This feature is fundamental if we want to use this WF to describe chemical reactions and perform large-scale simulations, with a size consistent behavior at large distances. The importance of the variational optimization of the WF is particularly evident in this small molecule. With the standard approach, by applying DMC to a SD taken by DFT (here obtained with Purdue and Zunger LDA^56^), a level crossing in the occupation of the π MOs occurs at around 3 bohr distance, above which the π bonding orbitals are only partially occupied. This implies clear artifacts in the DMC energies. We have verified that this level crossing is reproduced with a standard DFT-LDA calculation by Gaussian 16 A.03 revision^57^ and an almost converged basis set (the standard cc-pVQZ). The level crossing has also been observed in ref (58). In our variational optimization instead, we have verified that it is important to start at large distance with the WF predicted by LSDA, otherwise a sizably higher energy is obtained. This effect is reflected also by the sharp change of the projected *S*^2^ at around 3 bohr distance (see Figure 2), that could be compatible with an avoided crossing between two energy levels belonging to the same ^1^Σ_*g*_^+^ representation.^59^

### 3.2 Nitrogen

Nitrogen is in some sense similar to the carbon case: also its dimer is indeed a singlet formed by two large spin 3/2 atoms.

As we can notice from Figure 1 and Table 4, at the DMC level, the JsAGPu and JAGP are both exact within chemical accuracy. All our calculations compare with the exact result better than the JFVCAS solution. Surprisingly, at the VMC level, the binding energies calculated with JAGP, JsAGPs, and JAGPu are also very good.

**Table 4:** Nitrogen Energies^a^

	**nitrogen**		
	atom	molecule	binding
source	energy [H]	energy [H]	energy [eV]
JSD	–54.5628(1)^b^	–109.4520(5)^b^	8.88(1)^b^
JFVCAS		–109.4851(3)^b^	9.78(1)^b^
JsAGPs	–54.55794(6)	–109.4781(7)	9.856(3)
JAGPu	–54.55998(5)	–109.48155(7)	9.840(3)
JAGP	–54.56633(5)	–109.49226(7)	9.785(3)
JSD (DMC)	–54.57587(4)^b^	–109.5039(1)^b^	9.583(3)^b^
JFVCAS (DMC)		–109.5206(1)^b^	10.037(3)^b^
JsAGPs (DMC)	–54.5765(1)	–109.5164(2)	9.88(1)
JAGPu (DMC)	–54.5767(3)	–109.5140(2)	9.81(1)
JAGP (DMC)	–54.57709(9)	–109.5192(1)	9.933(6)
Fermi net	–54.58882(6)^c^	–109.5388(1)^c^	9.828(5)^c^
estimated exact	–54.5892^d^	–109.5427^e^	9.908(3)^e^

a The JsAGPs, JAGPu, and JAGP results are calculated with an optimized ccpVTZ basis set.

b Reference (17).

c Reference (5).

d Reference (54).

e Reference (55).

We remark that a very powerful method, as the recently proposed Fermi net^5^ (a neural network-based WF), cannot reach the same precision in the binding energy even if the total energies of the molecule and atom are the best available ones. This clearly shows that all our ansatzs allow a remarkable cancellation of errors, when computing the total energy differences between the molecule and the two independent atoms.

**Figure 3:**
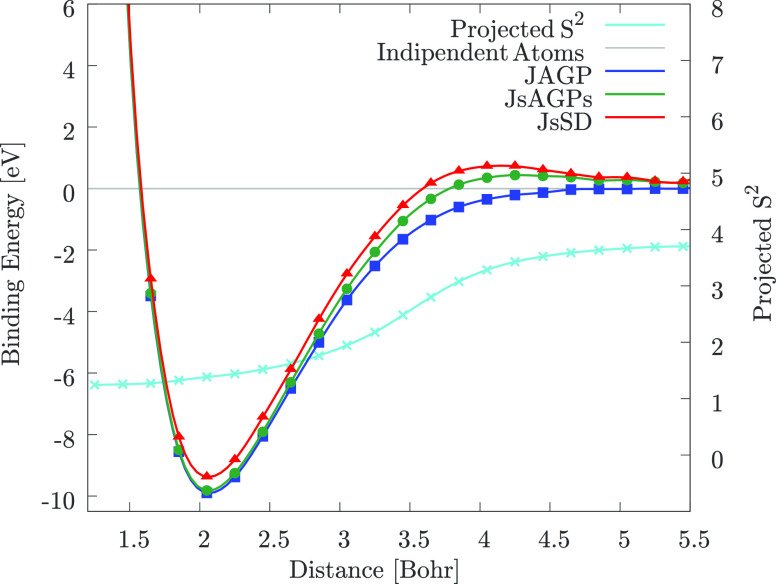
DMC energy dispersion of the nitrogen dimer: only the JAGP appears to be perfectly size consistent, thus recovering the energy and the expectation value of the *S*^2^ operator of two isolated atoms at large interatomic distance. At bond distance, however, the nitrogen atoms have a smaller value of *S*^2^, in contrast to what observed for the carbon dimer. Lines are guides to the eye.

In this case, however, the difference between JAGP and JsAGPs/JAGPu is much smaller than in the previous case and should be related to a less important role of the spin fluctuations and also to a smaller magnetic moment of the atoms at equilibrium distance. By repeating the reasoning done for the carbon dimer, we can quantify the magnetic moment from the *S*^2^ value in the semi-infinite region separated by a plane perpendicular to the axis of the molecule and equidistant from the atoms. As shown in Figure 3, at bond distance, the *S*^2^ of the atom is much smaller than the one of an independent atom, and therefore, even if the nitrogen atom has a large spin, when it is forming a dimer it does not give rise to a strong antiferromagnetism.

Also in this case, it is important to notice that the JAGP solution is size consistent both in energy and spin. Despite the very good description at bond distance provided by the JsAGPs, we notice from Figure 3 that it is not perfectly size consistent. Within our approach, a fully consistent picture and a very accurate dispersion are possible only by means of the JAGP ansatz, that is able to work properly also in the strong correlation regime at large interatomic distance.

### 3.3 Oxygen

The oxygen is very different from the previous cases but nevertheless very interesting for different reasons. The oxygen dimer consists of two triplet atoms, but this time the molecule is a triplet. There are small atomic magnetic moments in the GS of the oxygen molecule, but the role of the magnetic interaction remains important, as shown by the application of the JAGP ansatz. In this case, it looks that the interaction of parallel spin electrons is particularly important, and this can be described by the JAGP ansatz more accurately than the corresponding JsAGPs and JAGPu ones, as discussed in the previous sections. Thus, we expect to recover with the JAGP some correlation that we miss when we simplify the ansatz by using the unpaired orbitals in the JsAGPs and in the JAGPu WFs.

As shown in Figure 1 and Table 5, at the DMC level, the energies obtained with the JAGP WF are extremely good even for the oxygen dimer. In this case, the correct description of the triplet pairing correlations, possible within the JAGP *ansatz*, appears to be fundamental. Indeed, the final result is so accurate that the binding energy is comparable to the one obtained with the multideterminant JFVCAS WF. It is even more surprising that the absolute energies of the atom and molecule are very close where not even better than the ones provided by the multideterminant expansion both at VMC and DMC levels. We have to point out, however, that within JFVCAS method, it is not possible to improve the JSD atom^17^ and that the binding energy slightly better than the JAGP one derives from the poorer quality of the atom rather than a better description of the molecule.

**Table 5:** Oxygen Energies^a^

	**oxygen**		
	atom	molecule	binding
source	energy [H]	energy [H]	energy [eV]
JSD	–75.0352(1)^b^	–150.2248(5)^b^	4.20(1)^b^
JFVCAS		–150.2436(2)^b^	4.713(8)^b^
JsAGPs	–75.0268(3)	–150.2372(6)	5.00(3)
JAGPu	–75.0339(3)	–150.2503(5)	4.97(3)
JAGP	–75.0346(2)	–150.2572(4)	5.11(2)
JSD (DMC)	–75.05187(7)^b^	–150.2872(2)^b^	4.992(7)^b^
JFVCAS (DMC)		–150.29437(9)^b^	5.187(5)^b^
JsAGPs (DMC)	–75.0518(3)	–150.2894(3)	5.06(2)
JAGPu (DMC)	–75.0519(3)	–150.2902(4)	5.06(2)
JAGP (DMC)	–75.05289(7)	–150.2942(1)	5.127(5)
estimated exact	–75.0673^c^	–150.3724^d^	5.241^d^

a The JsAGPs, JAGPu, and JAGP results calculated with an optimized ccpVTZ basis set.

b Reference (17).

c Reference (54).

d Reference (55).

**Figure 4:**
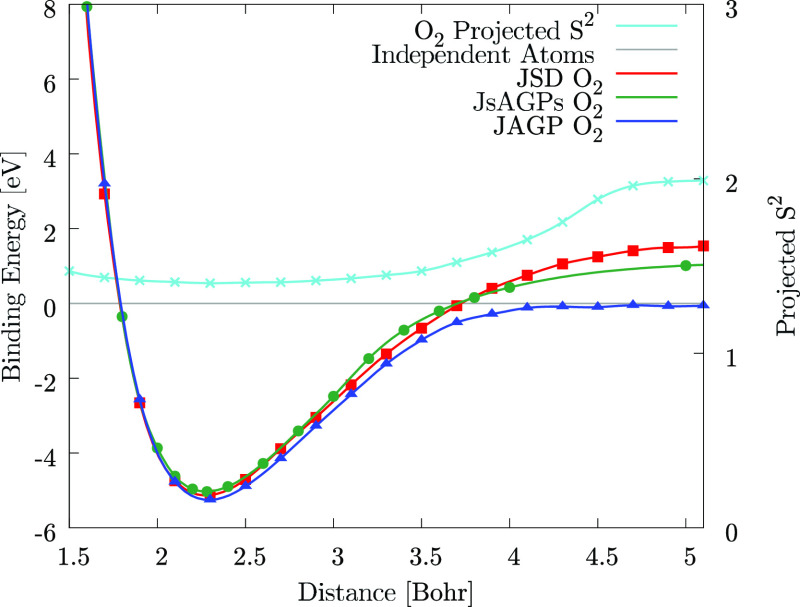
DMC energy dispersion of the oxygen dimer with the JAGP, JsAGP,^60^ and JSD (with the SD obtained from DFT calculations): at large distance, only the JAGP WF is size consistent. In the plot, also the expectation value of the projected *S*^2^ operator on the atoms for the JAGP that recovers the value of two isolated atoms at large distance. Lines are guides to the eye.

The problem of the size consistency for the oxygen dimer is absolutely nontrivial and even more complicated than the previous cases. Starting from bond distance, we have a molecule of spin one and fixed projection *S*_*z*_ = 1, but we have to recover the behavior of two independent atoms. This means that, by keeping the projection *S*_*z*_ = 1 constant, while separating the atoms far apart, we have to recover the correct atoms of spin one and thus we need to have one atom with the spin oriented in a direction perpendicular to the *z*-axis. This is impossible for the JsAGPs and the JAGPu but allowed by the JAGP, a remarkable and absolutely nontrivial feature of this WF. As we can see from Figure 4, at large distance, only with the JAGP the system recovers the energy and the spins of the independent atoms, showing that, by means of our advanced optimization tools, it is possible to dramatically change the WF up to the point of rotating completely the spin of an atom.

### 3.4 Benzene

The benzene molecule represents one of the most successful examples of the RVB theory with the carbon–carbon bonds resonating among several valence bond configurations, for example, Kekulé and Dewar. QMC methods are able to provide a very good description of this important molecule,^61,62^ and thus, it is interesting to check whether, with our new approach, we can obtain a very accurate result. In particular, in Table 6, we compare the results obtained by JSD, JAGP, JAGPu, and JsAGPs WF, showing that all results obtained with a pairing function (from JsAGPs to JAGP) provide a very good estimate of the absolute energies, noticeably improving the results of the JSD. Moreover, the corresponding atomization energies are extremely accurate at the DMC level, whereas the JSD largely overestimates it. It is finally interesting to notice that, even if there is a sizeable gain in terms of absolute energy with our best ansatz, that is, the JAGP, it is not clear why this systematic improvement does not sizeably affect the atomization energy, likewise this could be almost converged to the exact value. This might be in principle explained because, at present, the accuracy of the state of the art “estimated exact” calculation is probably not enough to establish an energy difference ≪0.1 eV. For instance, the ZPE has been estimated by DFT^63^ and some work is certainly necessary to clarify this issue, for example, by calculating the ZPE directly with QMC.

**Table 6:** Benzene Energies

	**benzene**		
	C atom^a^	molecule	atomization energy
source	energy[H]	energy[H]	energy[eV]
JSD	–37.8074(1)	–232.0261(3)	59.37(1)
JsAGPs	–37.82383(4)	–232.0805(3)	58.166(8)
JAGPu	–37.82651(5)	–232.0900(3)	57.986(8)
JAGP	–37.82921(4)	–232.1060(2)	57.982(7)
JSD(DMC)	–37.8299(1)	–232.1879(6)	60.09(2)
JsAGPs(DMC)	–37.8368(1)	–232.1947(6)	59.16(2)
JAGPu(DMC)	–37.8367(1)	–232.1943(6)	59.16(2)
JAGP(DMC)	–37.83751(9)	–232.1998(5)	59.18(2)
estimated exact	–37.8450^b^	–232.250(1)	59.32(2)^c^

a Calculated with the same basis set used for the benzene molecule.

b Reference (54).

c Reference (63).

We remark here that the JsAGPs description of the benzene molecule is already very accurate and it is not improved by the JAGP. This is probably due to the lack of any sizeable spin moment around any atom composing this molecule. Indeed, the *S*^2^ value calculated for the JAGP and JAGPu solutions are 0.032(1) and 0.0123(7), respectively, proving that any local magnetic moment is almost completely melted during the optimization, despite its nonzero initialization. We conclude therefore that in the benzene molecule, the spin fluctuations are not relevant and the use of the Pfaffian leads only to a marginal improvement of the total energy while the molecule is correctly described by a perfect singlet RVB ansatz given by the JsAGPs, in agreement with the classical RVB picture by L. Pauling.^64^

## 4 Conclusions

In this work we have proposed a new WF for QMC calculations given by the most general fermionic pairing function *ansatzs* in combination with a spin JF that provides a very rich description of the electronic correlation by means of a bosonic pairing function complementary to the fermionic one. With a computational cost comparable to a SD, we were able to improve not only the results achieved with a simple JSD but also with JsAGPs and JAGPu, reaching a level of accuracy comparable to the one obtained with the multideterminant JFVCAS WF. The powerful optimization techniques are probably the keys to explain the remarkable improvement we obtained with this WF, compared to previous attempts.^23,29^ In particular, we have shown that the JAGP *ansatz* provides a very accurate description of high spin atoms and their dimers and that it is size consistent. This should increase the number of possible applications, providing a reasonably accurate and computationally feasible tool for studying chemical reactions. The triplet correlations have proven to be necessary to take into account correctly the ZPE of the spin fluctuations that we can now correctly describe thanks to a physical and accurate setup obtained by orienting the atomic magnetic moments of the AGP in the direction perpendicular to the spin-quantization axis chosen for the JF. For this reason, we have obtained a very good description of the carbon and nitrogen dimers, remarkably even when the first molecule was found to be very poorly described by the JsAGPs and the JAGPu. Moreover, it is only thanks to the presence of the triplet correlations that we were able to improve the description of the oxygen dimer as a strongly correlated triplet molecule with a highly entangled spin interaction among the atoms. Comparison with other methods different from QMC is shown in App. A. Our QMC variational energy is much better than state of the art quantum chemistry methods that seem to be affected by strong basis set errors even when considering only energy differences. For instance, the total energy difference Δ*E* at *R* = 4.2 a.u. and *R* = 2.11 a.u. in Table 8 should be close to the estimated exact binding energy (i.e. ≃9.91 eV from ref (55)) at most weakly corrected by the residual dispersive interaction. Both DMRG and MRCI clearly miss more than 1 eV with the DZ basis, that is, Δ*E* ≃ 8.49 eV. In order to show more clearly that the discrepancy between our DMC results and DMRG and MRCI is actually an artifact of the small basis, we have carried out UCCSD(T) calculation both for small (Δ*E* = 8.6 eV) and large (Δ*E* = 9.55 eV) basis set, and, as expected, our calculation (Δ*E* = 9.63) is much more in agreement with the most accurate large basis set calculation. In any event, our binding energy for *N*_2_ (9.933 ± 0.006 eV) is surprisingly more accurate than the best state of the art calculation with CCSD(T) (9.73 eV from the Computational Chemistry Comparison and Benchmark DataBase^65^), implying that, most likely, our results should be considered to be the state art for the full dispersion curve of these small molecules.

**Table 7:** Carbon Energy Dispersion (Hartree)^a^

	numerical technique					
distance	JAGP (DMC)	DMRG	HCI	UCCSD-T_frozen_	UCCSD-T_full_	FCI
2.0787	–75.86652(3)	–75.76125^c^	–75.76701^d^	–75.76085	–75.78683	–75.7624^e^
2.2677	–75.90207(3)	–75.79924^c^	–75.80461^d^	–75.78450	–75.80878	–75.7987^e^
2.3480	–75.90456(3)	–75.80269^c^	–75.80786^b^^,^^d^	–75.78370	–75.80754	–75.8025^e^
2.4566	–75.90008(3)	–75.79937^c^	–75.80444^d^	–75.77928	–75.80247	–75.7993^e^
2.6456	–75.87825(4)	–75.77937^c^	–75.78460^d^	–75.76465	–75.78664	–75.7798^e^
3.0235	–75.81700(8)	–75.72405^c^	–75.72895^d^	–75.71762	–75.73765	–75.7243^e^
3.7794	–75.73649(8)	–75.64560^c^	–75.65043^b^^,^^d^	–75.62162	–75.63996	–75.6454^e^

a The JAGP results were obtained with the optimized ccpVDZ basis set (as explained in Section 2.1), the DMRG results with the ccpVQZ basis, the HCI with ccpV5Z basis set, the FCI with ccpVQZ basis set, whereas the UCCSD-T ones, both full- and frozen-core, are shown for ccpV5Z basis sets.

b Interpolated.

c Reference (59).

d Reference (66).

e Reference (67).

**Table 8:** Nitrogen Energy Dispersion (Hartree)^a^

	numerical technique				
distance	JAGP (DMC)	DMRG	MRCC	UCCSD-T (DZ)	UCCSD-T (5Z)
2.118	–109.51694(5)	–109.27833^c^	–109.27683^c^	–109.27652	–109.41303^b^
2.4	–109.46459(6)	–109.23838^c^	–109.23687^c^	–109.23202	–109.35926
2.7	–109.37935(6)	–109.16029^c^	–109.15895^c^	–109.14731	–109.26936
3.0	–109.29961(6)	–109.08619^c^	–109.08442^c^	–109.06570	–109.18331
3.6	–109.19745(6)	–108.99489^c^	–108.99272^c^	–108.97982	–109.08833
4.2	–109.16376(7)		–108.96471^c^	–108.96002	–109.06204

a The JAGP results were obtained with the optimized ccpVDZ basis set (as explained in Section 2.1), the DMRG and MRCC results with the ccpVDZ basis, whereas the corresponding UCCSD-T ones are shown also for a much larger basis (ccpV5Z), resulting in much better agreement with the present DMC results.

b Interpolated.

c Reference (68).

Finally we demonstrated that for the benzene dimer, the JAGP is able to provide a very accurate atomization energy, though it is not clear in this case whether the triplet correlations are crucial for a highly accurate calculation. However, it is important to highlight that the accuracy in the binding energy is always much better than the accuracy in the total energy and that therefore there exists always a remarkable cancellation of errors in the total energy differences. This feature indeed is fundamental for a compact *ansatz* like the JAGP, and it challenges other very expensive highly correlated methods, even when these are able to achieve almost exact total energies as it was the case for the Fermi net approach to the nitrogen dimer.

The relatively low computational cost of QMC combined with powerful optimization techniques, allowing a reasonably large number of variational parameters, makes this approach ideal for studying systems even much larger than the ones considered in this work. Indeed, we believe that the paradigm presented in this paper could represent in the future a very powerful tool to investigate the electronic structure of interesting chemical compounds and physical systems where the spin interaction may play an important role, that in turn may be a number much larger than previously believed, as we have presented here the C_2_ molecule as the very first and remarkable example of an antiferromagnetic chemical bond.

## Figures and Tables

**Figure 5:**
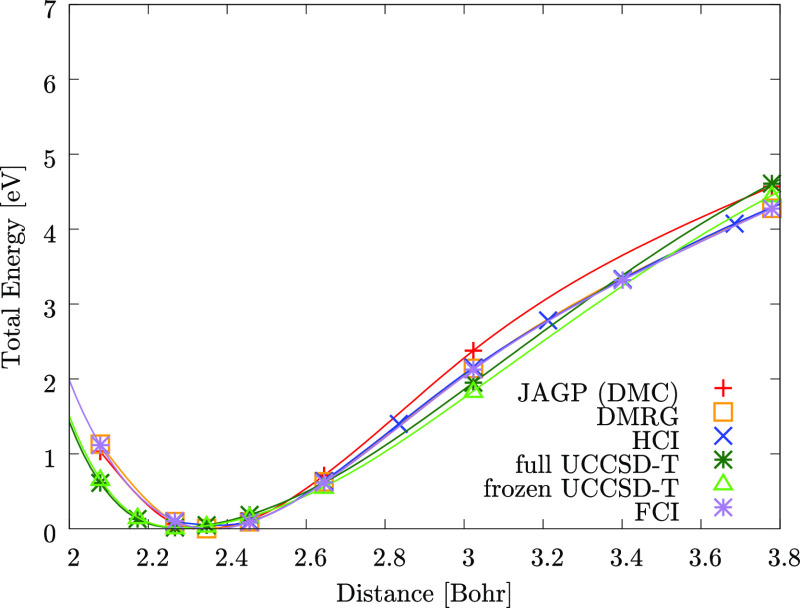
Energy dispersion of the carbon dimer calculated with JAGP (DMC), UCCSD-T (ccpV5Z), DMRG,^59^ HCI,^66^ and FCI.^67^ Lines are guides to the eye.

## Appendix

### Energy Dispersion Comparison

Comparison between the energy dispersion calculated with DMC JAGP WF and unrestricted single reference coupled cluster (UCCSD-T) with ccpVDZ and ccpV5Z basis sets. We further compared the carbon energy dispersion with DMRG, heat-bath configuration interaction (HCI) and full configuration interaction (FCI) from the literature,^59,66,67^ and the nitrogen dispersion with multireference coupled cluster (MRCC) and DMRG.^68^ UCCSD(T) calculations were performed using Gaussian 16 A.03 revision with the counterpoise correction, with the frozen-core approximation and the full-core correlation.^57^Table 7 and Figure 5 show that there are significant discrepancies between different methods in the carbon dimer dispersion curve at large distances. However, one has to consider that even in a quadruple zeta basis, the binding energy *D*_e_ = 6.22 eV^67^ is about 6 mH lower than the estimated exact one and therefore if we reference all curves at the bond length minimum energy, as reported in the mentioned figure, a method that is supposed to be weakly dependent on the basis, as our DMC, should be slightly higher in energy at large distance, provided it remains close to the exact dispersion energy curve. Moreover, there may be sizeable corrections due to the frozen-core approximation employed by DMRG, HCI, and FCI. We have indeed verified that they are non-negligible in the UCCSD-T calculation, implying that core–valence interaction can lead to a further nonparallelity error of about 3 mH (see Figure 5). Core–valence interaction is considered in our DMC calculations simply because, within this technique, it is not possible to employ the frozen-core approximation. Nevertheless, it is clear that our results may have some error, but it is remarkable that if we use the corresponding energy values for computing the ZPE of the dimer, we find excellent agreement with the experimental value, given by 0.1146 eV.^69^ Indeed, the ZPE calculated values, using a standard fit with a quartic polynomial close to the equilibrium distance, are 0.1153(6), 0.108, 0.106, 0.112, 0.114, and 1133(3) eV for our DMC, UCCSD-T full-core, UCCSD-T frozen-core, DMRG, HCI, and FCI, respectively. In summary, by taking into account all possible sources of error, we believe that our results are in reasonable agreement with the expected “exact result” converged in the complete basis set limit and with full core–valence interaction taken into account. Indeed, we believe that only a more direct comparison with experiments or a full-core FCI/DMRG or HCI extrapolated to the complete basis set limit can further improve the accuracy of the dispersion curve.

### Diagonalization of a Skew Symmetric Generally Complex Matrix λ

Here, we discuss a general procedure to transform a generic complex antisymmetric matrix into a canonical Youla form that represents the equivalent of the standard diagonalization of Hermitian matrices. This is obtained by means of an appropriate unitary matrix *U* defined by an orthonormal set of states that we will address in the following MOs.

Given a *N̅* × *N̅* antisymmetric matrix λ, our goal is to identify a set of *p* paired states {(ϕ_*j*_^1^, ϕ_*j*_^2^)} of orthonormal MOs, such that *p* ≤ *N̅* is even and

55

56

where the LHS of the above equations indicate standard matrix vector products, with shorthand notations adopted also in the remaining part of this Appendix. In this basis, we can write any skew-symmetric matrix λ in the canonical Youla form

57

using only *p*/2 strictly positive parameters *a*_*j*_. These ones play the same role of the eigenvalues for an ordinary Hermitian matrix and henceforth we will use this name for them, even if the matrix λ_MO_ is not diagonal but represents the simplest nonvanishing skew-symmetric matrix.

The transformation of the original matrix λ to the corresponding canonical Youla form by means of an appropriate unitary transformation λ = *U**λ_MO_*U*^†^ provides us also a very simple way to regularize the matrix λ, as discussed in the main text. In the case of odd *N̅*, it will be shown later that there exists always an eigenvector of λ with a vanishing eigenvalue, but the decomposition remains possible as λ_MO_ will contain at least one vanishing row and corresponding column. Here, we define that an eigenvector is singular if it corresponds to a vanishing eigenvalue as in the odd *N̅* case.

It would be ideal for this calculation to use a very robust and stable diagonalization routine to maintain machine accuracy for the MOs. Unfortunately these routines are not commonly available for antisymmetric matrices and thus several mathematical transformations are necessary to map our task to a sequence of more commonly used or at least easily available algorithms.

A generic *N̅* × *N̅* antisymmetric matrix is written in the following way

58

The first step is to transform λ in a tridiagonal antisymmetric real matrix. This operation is implemented in the subroutine zsktrd (dsktrd) contained in the PFAPACK library.^49^ The use of the Householder algorithm allows us to decompose the generic matrix λ as

59

where *U*_1_ is the unitary transformation matrix output of the algorithm, while λ_Tr_ is a tridiagonal real antisymmetric matrix written in the standard tridiagonal form

60

Thus, we can multiply the matrix λ_Tr_ for the imaginary unit *i*, yielding a more conventional tridiagonal Hermitian matrix λ_*iH*_, defined by purely imaginary matrix elements.

We highlight that it is possible to map the matrix λ_*iH*_ into a real Hermitian matrix via a unitary transformation and use the appropriate LAPACK routine for its fast diagonalization. This procedure is well known and will be discussed later.

At this point, we can use the spectral theorem for Hermitian matrices to decompose the matrix λ_*iH*_ = ψλ_diag_ψ^†^, where λ_diag_ is a diagonal matrix containing in its diagonal part the real eigenvalues *a*_*i*_ of λ_*iH*_, and ψ is the unitary matrix, where each column is given by the eigenvector in the principle complex, corresponding to each eigenvalue, in the chosen order. This decomposition implies:

61

However, because the matrix ψ is generally complex and ψ^†^ ≠ ψ^*T*^, some manipulation is necessary if we want to satisfy the skew-symmetry property of λ in an easy and transparent way.

If we consider one eigenvector ψ̅_*j*_ associated to an eigenvalue *a*_*j*_ > 0, we have

62

the complex conjugate of this expression is

63

where we have used that both λ_Tr_ and the eigenvalues *a*_*j*_ are real. This means that if ψ̅_*j*_ is an eigenvector of λ_*iH*_ relative to the eigenvalue *a*_*j*_, then ψ̅_*j*_^*^ is an eigenvector corresponding to the eigenvalue −*a*_*j*_ and thus orthogonal to ψ̅_*j*_ because of the orthogonality between eigenvectors of an Hermitian matrix corresponding to different eigenvalues ± *a*_*j*_. We can thus easily verify, by using the relations given in eqs 62 and 63, the following simple equations

64

65

aIn this way, we can define pairs of real orthogonal vectors  and  such that

66

67

Once we have identified all pairs corresponding to all positive eigenvalues *a*_*j*_ > 0, we can write the unitary matrix ψ̅, that is now real, by adding the remaining eigenvectors (that can be also chosen real, as shown in subsection Singular Eigenvectors) with vanishing eigenvalues in the remaining rightmost columns. In this way, we can finally define a unitary real matrix ψ̅ yielding λ_Tr_ = −*i*λ_*iH*_ = ψ̅λ_MO_ψ̅^*T*^, where λ_MO_ is defined in eq 57 and therefore by using eq 59

68

which represents the desired decomposition because the product of two unitary matrices *U** = *U*_1_^*^ψ̅ remains a unitary matrix and its transponce *U*^†^ coincides with ψ̅^*T*^*U*_1_^†^, yielding λ = *U**λ_MO_*U*^†^, that is the purpose of this Appendix.

### Triangular Hermitian Matrices: A Mapping from Imaginary to Real

In order to use the LAPACK routines for diagonalization, we have to map the tridiagonal fully imaginary Hermitian matrix λ_*iH*_, defined only (the diagonal elements are zero to fulfill Hermitianity) by its upper diagonal elements *ib*_*j*_ with *b*_*j*_ real for *j* = 1, 2, ..., *N̅* – 1, into a tridiagonal real symmetric matrix λ_R_. We can implement this mapping by applying a unitary transformation to the matrix λ_*iH*_. For this purpose, we introduce the following transformation described by the matrix *U*_2_

69

The matrix *U*_2_ is a complex diagonal matrix defined as

70

The explicit calculation of the right-hand side of the eq 69 gives

71

By setting the imaginary units ± *i* = exp(±*i*π/2) (when not exponentiated in the previous equation), we can easily impose that all phase factors cancel in all the corresponding matrix elements of λ_R_ with the choice

72

that therefore implies that λ_R_, with the above definition, is a real symmetric matrix. At this point, we can diagonalize the matrix λ_R_ by means of a real unitary matrix *U*_R_, that is the output of a standard LAPACK diagonalization routine of tridiagonal real matrices (e.g., dstevx for double precision arithmetic). In this way, λ_*iH*_ can be diagonalized as λ_*iH*_ = *U*_2_*U*_R_λ_diag_*U*_R_^*T*^*U*_2_^†^, where λ_diag_ is a diagonal matrix containing the corresponding eigenvalues of the LAPACK diagonalization.

### Singular Eigenvectors

Within this formulation, it is also particularly easy to compute all the real singular eigenvectors of λ_*iH*_ corresponding to the possible vanishing eigenvalues. They were used in this Appendix to complete the columns of the unitary real matrix ψ̅. From the outcome of the previous subsection, any eigenvector ϕ_*k*_^*j*^ of λ_*iH*_ can be obtained by applying the diagonal matrix *U*_2_ to a real eigenvector ϕ̅_*k*_^*j*^ of λ_R_, namely, ϕ_*k*_^*j*^ = ϕ̅_*k*_^*j*^ exp(−*i*π/2(*k* – 1)), implying that even *k*-components are purely imaginary and odd *k*-components are purely real. Then, it is obvious to realize that ϕ̅_*k*_^*j*^ corresponds to a singular eigenvector of λ_R_, and also  and  [and therefore also ] correspond to singular eigenvectors or at most null vectors (not both) of λ_*iH*_ because this matrix is purely imaginary, and the complex conjugation of a singular eigenvector is again a singular eigenvector by eqs 62 and 63 with *a*_*j*_ = 0.

Then, it follows that all orthogonal eigenvectors ϕ̅^*j*^ (output of dstevx) corresponding to the zero eigenvalues of the matrix λ_R_ can be used to define the real singular eigenvectors corresponding to the matrix λ_*iH*_, that is

73

that are explicitly real and orthogonal to each other because ∑_*k*_ϕ̃_*k*_^*j*^ϕ̃_*k*_^*l*^ = ∑_*k*_ϕ̅_*k*_^*j*^ϕ̅_*k*_^*l*^ = δ_*l*,*j*_. They are also orthogonal to all the other pairs of non-singular eigenvectors because of the orthogonality property of eigenvectors of an Hermitian matrix λ_*iH*_ that we have already used in the previous section.
